# Water immersion for post incident cooling of firefighters; a review of practical fire ground cooling modalities

**DOI:** 10.1186/s13728-015-0034-9

**Published:** 2015-09-30

**Authors:** Matt Brearley, Anthony Walker

**Affiliations:** National Critical Care and Trauma Response Centre, Level 8 Royal Darwin Hospital, Rocklands Drive, Tiwi, NT 0810 Australia; Discipline of Sports Studies, Faculty of Health, UC Research Institute for Sport and Exercise, University of Canberra, Canberra, ACT 2601 Australia; Australian Capital Territory Fire and Rescue, Amberley Avenue, Fairbairn Business Park, Majura, ACT 2609 Australia

**Keywords:** Cooling, Core body temperature, Firefighter, Forearm, Heat, Immersion, Water

## Abstract

Rapidly cooling firefighters post emergency response is likely to increase the operational effectiveness of fire services during prolonged incidents. A variety of techniques have therefore been examined to return firefighters core body temperature to safe levels prior to fire scene re-entry or redeployment. The recommendation of forearm immersion (HFI) in cold water by the National Fire and Protection Association preceded implementation of this active cooling modality by a number of fire services in North America, South East Asia and Australia. The vascularity of the hands and forearms may expedite body heat removal, however, immersion of the torso, pelvis and/or lower body, otherwise known as multi-segment immersion (MSI), exposes a greater proportion of the body surface to water than HFI, potentially increasing the rates of cooling conferred. Therefore, this review sought to establish the efficacy of HFI and MSI to rapidly reduce firefighters core body temperature to safe working levels during rest periods. A total of 38 studies with 55 treatments (43 MSI, 12 HFI) were reviewed. The core body temperature cooling rates conferred by MSI were generally classified as ideal (n = 23) with a range of ~0.01 to 0.35 °C min^−1^. In contrast, all HFI treatments resulted in unacceptably slow core body temperature cooling rates (~0.01 to 0.05 °C min^−1^). Based upon the extensive field of research supporting immersion of large body surface areas and comparable logistics of establishing HFI or MSI, it is recommended that fire and rescue management reassess their approach to fireground rehabilitation of responders. Specifically, we question the use of HFI to rapidly lower firefighter core body temperature during rest periods. By utilising MSI to restore firefighter T_c_ to safe working levels, fire and rescue services would adopt an evidence based approach to maintaining operational capability during arduous, sustained responses. While the optimal MSI protocol will be determined by the specifics of an individual response, maximising the body surface area immersed in circulated water of up to 26 °C for 15 min is likely to return firefighter T_c_ to safe working levels during rest periods. Utilising cooler water temperatures will expedite T_c_ cooling and minimise immersion duration.

## Background

Emergency firefighting requires firefighters to wear heavy, multilayered, impermeable personal protective clothing (PPC). While providing protection from radiant heat, flame and hazardous chemicals, PPC creates an uncompensable environment that actively limits dissipation of body heat [1]. The combination of hot ambient temperatures and intense physical work leads to increases in core body temperature (T_c_). Firefighting and rescue simulations, conducted with participants wearing PPC, resulted in individual peak T_c_ of 39.6–40.9 °C [2–5], synonymous with exertional heat exhaustion in laboratory settings [6, 7]. In the field, high T_c_ would likely limit work output due to an acute impact on physiological [8] and psychological function [9]. High T_c_ is also likely to be coupled with adverse immune and inflammatory activity [10–12]. To mitigate the risk of high T_c_ precipitating fatigue and ultimately exertional heat illness, firefighters rotate between work tasks and rest breaks where feasible. Since firefighters are regularly tasked with re-entering fire scenes or are redeployed to other emergency work tasks, breaks can be restricted to less than 20 min between work bouts. In that time, to maintain operational readiness, firefighters are required to replenish their equipment and PPC prior to engaging in rehabilitative practices such as rehydration and cooling [13]. Hence, the development of rehabilitation protocols, inclusive of rapid and effective cooling modalities, is a pre-requisite to maximising the health, safety and performance of firefighters during sustained emergency responses.

To achieve rapid post-incident cooling of firefighters, a variety of modalities including ice vests [14–16], misting fans [2, 4], iced towels [17], crushed ice ingestion [2, 5], cool intravenous fluids [18], hand and/or forearm water immersion [4, 19–21] and whole or lower body water immersion [2, 5] have been investigated. Of these methods, the National Fire and Protection Association (NFPA), the peak advisory body in the USA recommends hand and forearm immersion (HFI) in cold water and use of misting fans as active cooling modalities for use in firefighting settings [22].

It is unclear how many fire services have implemented HFI based upon the NFPA recommendation, however, HFI is documented within rehabilitation guidelines of fire services in North America, South East Asia and Australia. The recommendation of HFI for post-incident cooling was based upon the research of Selkirk et al. [4]. That study demonstrated that HFI (~17 °C) was more effective in lowering core body temperature during a rest phase than a commercially available misting fan, or passive cooling in the form of seated rest in the shade (35 °C, 50 % relative humidity). Despite support for HFI, an emerging field of research in Australia has demonstrated the effectiveness of lower body [5] and mid sternum depth water immersion [2] to lower T_c_ of urban firefighters following simulated work tasks in the heat. Immersion of the torso, pelvis and/or lower body, referred to as multi segment immersion (MSI), exposes a greater proportion of the body surface to water than HFI, potentially increasing the rates of cooling conferred. However, the vascularity of the hands and forearms may expedite heat removal to offset the limitation of immersing a small surface area. Hence, it is currently unclear whether the outcomes of HFI and MSI make either a preferable option for field cooling of firefighters. Therefore, this review aims to establish the efficacy of water immersion of the hands and/or forearms, and immersion of multiple body segments in firefighting settings to rapidly reduce T_c_ to safe working limits of less than 38.0 °C [23].

## Methodology

A computerised search of Pubmed, Google Scholar and SportDiscus was finalised in July 2015, using the following search terms: cold water, cold water immersion, cooling, cooling modality, cryotherapy, firefighter, forearm, forearm cooling, hand cooling, heat stress, heat stroke, ice bath, ice water immersion, physical activity, rehabilitation, torso and water immersion. Additional published studies known to the authors, and reference list searches of the retrieved studies complemented the initial examination. Inclusion in the present review was assessed against the following research criteria prior to data analysis; original research on human participants, body heat storage induced by physical activity, minimum pre-treatment T_c_ of 38.2 °C reflecting the lowest anticipated T_c_ during arduous emergency responses, minimum of five continuous minutes of immersion, provision of sufficient detail to reproduce methodology and valid T_c_ measurement. Specifically, those studies reporting tympanic temperature data were excluded due to lack of validity [24, 25].

For the purpose of this review, no distinction between hand and forearm immersion was made. It is acknowledged that the surface area of the hands (5 %) varies from the forearms (14 %) [26]. However, based upon similar logistics, cooling dynamics and the small number of studies meeting the inclusion criteria, hand and/or forearm immersion studies were pooled. Therefore, the abbreviation HFI collectively refers to cooling of any aspect of the lower arm and/or hand in water. In contrast, MSI refers to the immersion of any combination of the torso, pelvis and/or lower body.

To assess the effectiveness of HFI and MSI protocols, T_c_ cooling rate (°C min^−1^) criteria (Table 1) were adapted from [27]. The cooling rate classifications of McDermott et al. [27] were based on time to lower T_c_ analogous with exertional heat stroke (42.2 °C) to ~38.9 °C. Cooling rates of 0.079 °C min^−1^ or greater were deemed acceptable and T_c_ cooling of greater than 0.155 °C min^−1^ were recognised as ideal.

**Table 1:** Rate of core temperature cooling in individuals from 42.2 to ~38.9 °C

Cooling category	T_c_ cooling rate
Ideal (<20 min)	>0.155 °C min^−1^
Acceptable (20–40 min)	0.079–0.154 °C min^−1^
Unacceptable (>40 min)	<0.078 °C min^−1^

Cooling categories represent desired time for cooling [27]

This review reports findings from studies that assessed cooling of non-symptomatic individuals in addition to exertional heat stroke sufferers. In some cases, the T_c_ prior to the use of active cooling interventions was lower than the target T_c_ of 38.9 °C cited by McDermott et al. [27]. To ensure firefighters do not exceed the upper working T_c_ limit of 39.0 °C [23], we proposed that T_c_ up to 38.0 °C prior to fire scene re-entry be considered appropriate in healthy, medically monitored and heat acclimatised firefighters. With an anticipated maximal rehabilitation time of 20 min, adjustments to the cooling rates of McDermott et al. [27] to improve specificity for firefighter settings are detailed by (Table 2). Modalities conferring T_c_ cooling rates of less than 0.07 °C min^−1^, exclusive of afterdrop are likely to require durations beyond 15 min, providing insufficient cooling of firefighters prior to their re-entry to fire scene or delaying their redeployment.

**Table 2:** Proposed rates of cooling for firefighting operations to allow for the rapid safe re-entry of firefighters to emergency incidents

Classification	T_c_ cooling rate
Ideal (<10 min)	>0.10 °C min^−1^
Acceptable (10–15 min)	0.07–0.10 °C min^−1^
Unacceptable (>15 min)	<0.07 °C min^−1^

The following descriptive statistics were reported for studies meeting the inclusion criteria; ambient temperature, water temperature, immersion duration, immersion depth, pre-treatment T_c_, and T_c_ cooling rate (°C min^−1^). Reported height and mass measurements in each study were used to calculate body mass index (BMI) [28] and body surface area (BSA) [29].

## Results

### Included studies

This review identified 43 treatments utilising MSI (Table 2) and 12 HFI treatments that met the inclusion criteria. Measurement of T_c_ was from the rectum or gastrointestinal tract and research was predominantly conducted in laboratories, with 11 treatments applied in field settings [2, 3, 5, 20, 21, 30–34]. Participants included firefighters, athletes and individuals presenting with exertional heat stroke or exhaustion in emergency medical settings. Water temperatures between 10 and 24.9 °C were utilised for HFI and participants had similar pre-treatment T_c_ (38.2–39.0 °C) and immersion durations (10–30 min). Conversely, the MSI studies were more heterogeneous in terms of water temperature (1–26 °C), immersion time (5–42 min), BSA immersed and mean pre-treatment T_c_ (38.2–41.7 °C). Immersions generally commenced within 5 min of exercise cessation.

The rate of T_c_ cooling for MSI ranged from ~0.01 to 0.35 °C min^−1^ (Table 3) and were generally considered to be ideal (n = 23) or acceptable (n = 8). Conversely, T_c_ cooling rates mediated by HFI ranged from ~0.01 to ~0.05 °C min^−1^ (Table 3), with all HFI and 12 MSI treatments resulting in unacceptable T_c_ cooling rates.

**Table 3:** Descriptive data for MSI studies meeting the inclusion criteria

Cooling rate	n	Age (years)	T_a_ (°C)	Mins to treatment	T_w_ (°C)	Duration (mins)	BMI (kg m^2^)	BSA (m^−2^)	T_c_ site	Pre T_c_	Δ T_c_ (°C min^−1^)	Immersion depth	References
Unacceptable	9M	24	20.0	0	20	25	24.5	2.0	GI	38.6	~0.01	Mid-sternum	[35]
	9M	29	18.0	5	8	15	21.7	1.8	GI	38.3	~0.03	Lower body	[36]
	13M	39	29.5	0	25	30	27.6	2.1	GI	38.5	~0.04^b^	Mid-sternum	[2]
	5M	25	NR	NR	10–12	30	26.2	2.0	R	38.8	0.04	Torso	[37]
	9M/7F	24	22.2	NR	14	30	22.3	2.0	R	38.7	~0.04^b^	Neck out	[30]
	9M	21	NR	10	10	20	23.5	2.0	GI	38.4	~0.04	Mid-sternum	[38]
	10M	27	24.0	25	14.3	20	23.8	2.0	R	38.2	~0.04	Mid-sternum	[39]
	12M	29	24.0	25	14.3	20	NR	NR	R	38.6	~0.04	Mid-sternum	[40]
	9M	29	18.0	5	15	15	21.7	1.8	GI	38.4	~0.05	Lower body	[36]
	12M	29	24.0	25	14.3	10	NR	NR	R	38.6	~0.05	Mid-sternum	[40]
	12M	29	24.0	25	14.3	5	NR	NR	R	38.6	~0.06	Mid-sternum	[40]
	10M	29	22.0	5	26	~42	26.8	2.1	R	40.0	0.06^a^	Mid-sternum	[41]
Acceptable	9M	19	NR	NR	25	30	19.6	1.6	R	38.5	~0.07	Neck out	[42]
	10M	29	23.0	5	14	15	NR	NR	R	38.8	~0.07	Mid-sternum	[43]
	12M	32	NR	NR	14	15	22.1	1.9	R	38.5	~0.08	Neck out	[44]
	10M	35	NR	5	14	15	24.0	2.0	R	38.6	0.08	Mid-sternum	[45]
	26M	39	19.7	10	15	15	25.8	2.0	GI	38.9	0.09^b^	Lower body	[5]
	10M	34	32.8	5	15	15	24.2	2.0	R	38.5	~0.09	Neck out	[46]
	10M	29	22.0	5	26	~25	24.9	2.0	R	40.0	0.10^a^	Mid-sternum	[41]
	8M	23	20-22	5	26	26	21.2	2.0	R	40.1	0.10^a^	Neck out	[47]
Ideal	4M	23	25.0	5	23.5	6	26.1	1.9	R	39.2	~0.11^a^	Mid-sternum	[48]
	6M/4F	22	25.0	1.5	2	16	22.9	1.8	R	39.6	0.14	Neck out	[49]
	3M/4F	22	25.7	2.7	14	17	23.6	1.8	R	39.8	0.15^a^	Neck out	[50]
	14M/3F	28	28.9	2-4	5.2	12	21.1	1.9	R	39.5	0.16^b^	Torso	[31]
	14M/3F	28	28.9	2-4	14	12	21.1	1.9	R	39.6	0.16^b^	Torso	[31]
	12M/3F	28	29.5	2-4	14	12	21.1	1.9	R	39.5	0.16^b^	Torso/upper legs	[32]
	10M	29	22.0	5	2	~16	26.8	2.1	R	40.0	0.16^a^	Mid-sternum	[41]
	8M	30	40.0	40	2	~12.5	24.6	2.0	R	39.6	0.17^a^	Mid-sternum	[51]
	8M	30	40.0	20	2	~14	24.6	2.0	R	39.9	0.17^a^	Mid-sternum	[51]
	4M	22	25.0	5	11.7	6	23.9	1.9	R	39.8	~0.18^a^	Mid-sternum	[48]
	8M	23	20-22	5	14	13.9	21.2	2.0	R	40.1	0.18^a^	Neck out	[47]
	3M/4F	22	25.7	2.7	8	15	23.6	1.8	R	39.8	0.19^a^	Neck out	[50]
	3M/4F	22	25.7	2.7	20	18	23.6	1.8	R	39.8	0.19^a^	Neck out	[50]
	12M/3F	28	29.5	2-4	5.2	12	21.1	1.9	R	39.5	0.20^b^	Torso/upper legs	[32]
	13M/1F	35	24.4	4-8	1–3	16	NR	NR	R	41.7	0.20^b^	Torso/upper legs	[33]
	9M	26	NR	NR	8	13.1	26.7	2.1	R	39.9	0.20	Mid-sternum	[34]
	8M	30	40.0	5	2	~12.5	24.6	2.0	R	40.1	0.21^a^	Mid-sternum	[51]
	131M/81F	32	23.3	NR	~10	NR	NR	NR	R	41.4	0.22^b^	Torso/upper legs	[52]
	8M	27	NR	NR	8	12.8	22.9	1.9	R	40.1	0.23	Mid-sternum	[34]
	7M/2F	26	~29.0	6.3	2	~6	24.6	1.9	R	39.6	0.26^a^	Mid-sternum	[53]
	10M	29	22.0	5	2	~9	24.9	2.0	R	40.0	0.27^a^	Mid-sternum	[41]
	18M	22	39.9	NR	~10	6.2	25.7	2.0	R	39.5	0.28^a^	Neck out	[54]
	3M/4F	22	25.7	2.7	2	9	23.6	1.8	R	39.8	0.35^a^	Neck out	[50]

*n* number of participants, *M* male, *F* female, *T*_*a*_ ambient temperature, *T*_*w*_ water temperature, *R* rectum, *GI* gastrointestinal tract, *NR* not reported

^a^circulated bath

^b^field treatment

### Ideal T_c_ cooling rates

The highest T_c_ cooling rate of 0.35 °C min^−1^ was reported when participants immersed ~93 % of their body surface area in circulated 2 °C water [50]. Water temperature was a factor in the effectiveness of the cooling protocols employed. Specifically, water immersion at 2 °C resulted in superior T_c_ cooling rates compared with 8 °C (0.19 °C min^−1^), 14 °C (0.15 °C min^−1^) and 20 °C (0.19 °C min^−1^) water. However, all temperatures assessed by Proulx et al. [50] were within the ideal cooling range, and of the 23 treatments producing T_c_ cooling rates deemed to be ideal, Proulx et al. [50] and Lee et al. [48] represented the only treatments utilising a water temperature greater than 14 °C. The 10 treatments that lowered T_c_ by a minimum of 0.20 °C min^−1^, utilised a water temperature range of 1 to ~10 °C, demonstrating the utility of cold water to rapidly lower T_c_. A range of immersion depths resulted in rapid T_c_ cooling. Specifically, ideal T_c_ cooling rates were achieved by immersion to depth of mid sternum [34, 41, 48, 51, 53], immersion of the torso without lower body cooling [31], torso and thigh cooling [32, 33, 52] and ‘neck out’ immersion in circulated water baths [47, 50, 54].

To achieve ideal cooling rates, water immersion commenced within 8 min of exercise cessation with the exception of [51]. The duration of immersion for the ideal cooling rate treatments was generally 12–18 min [31–34, 41, 47, 49–51] while six treatments were conducted using cooling durations of approximately 10 min or less [41, 48, 50, 52, 54]. Pre-treatment T_c_ of ideal cooling rate studies were reflective of sustained body heat storage synonymous with a hyperthermic state (T_c_ 39.5–41.7 °C) [31–34, 41, 47–54]. Armstrong et al. [44] reported the highest mean pre-treatment T_c_ of 41.7 °C (individual peak 43.2 °C) from a cohort of 14 heat stroke symptomatic athletes. A mean immersion time of 16 min in 1–3 °C water returned T_c_ to an asymptomatic range, achieving a T_c_ cooling rate of 0.20 °C min^−1^ in an ambient temperature of 24.4 °C [44]. Ambient conditions for the other studies reporting ideal cooling rates were considered temperate to warm (20.0–29.5 °C). With the exception of [34, 41, 48, 54], the participants in the studies reporting ideal cooling rates were classified within the ‘normal’ BMI range (21.1–24.9 kg m^−2^).

Overall, ideal T_c_ cooling rates were generally achieved by immersion of participants with substantially elevated pre-treatment T_c_ for 12–18 min, in water temperatures up to 14 °C.

### Acceptable T_c_ cooling rates

Of the eight acceptable treatments, five immersions commenced up to 5 min following end of exercise protocol. Of the remaining three treatments, immersions were 10 min post exercise [5] or not reported [42, 44]. Given a need for firefighters to replenish their equipment and, increasingly engage in decontamination procedures prior to cooling, a delay of up to 10 min is likely to be seen in firefighting settings. Studies with acceptable T_c_ cooling rates generally employed higher water temperatures than ideal T_c_ cooling rate treatments. Five of the eight treatments resulting in acceptable T_c_ cooling rates utilised 14 or 15 °C water immersion [5, 43–46]. Three treatments employed temperate water immersion (25–26 °C) to achieve T_c_ cooling rates of ~0.07 to 0.10 °C·min^−1^ [41, 42, 47]. Despite similar water temperature (14 °C) and immersion duration (15–17 min), Robey et al. [43] reported a T_c_ cooling rate (~0.07 °C min^−1^) approximately half that of Proulx et al. [50]. In order to overcome the limiting factor of warmer water temperatures, ideal cooling rates were achieved by circulating water during immersion, with water depth to mid sternum or neck [41, 47].

Studies reporting cooling rates in the acceptable range, generally used immersion to depths of mid sternum or neck [41–47]. Walker et al. [5] was the sole study classified as acceptable that exclusively utilised pelvic and lower body immersion. Immersion duration ranged from 15 to 30 min, with five treatments utilising 15-min immersions [5, 43–46]. Pre-treatment T_c_ (38.5–40.1 °C) was generally lower than the ideal T_c_ cooling rate studies. Ambient conditions for studies reporting acceptable cooling rates were considered temperate to hot (19.7–32.8 °C). As for the ideal T_c_ cooling rate studies, the acceptable T_c_ cooling rate cohorts were classified within the ‘normal’ BMI range (19.6–24.9 kg m^−2^), with the exclusion of one cohort (25.8 kg m^−2^) [5].

Overall, studies reporting T_c_ cooling rates in the acceptable range generally utilised immersion to mid-sternum or neck depth in cool to temperate water (15–26 °C) for participants with pre-treatment T_c_ of 38.5–40.1 °C.

### Unacceptable T_c_ cooling rates

Twelve MSI and all 12 HFI cooling treatments were classified as delivering unacceptable T_c_ cooling rates. With the exception of Hausswirth et al. [35] and Dunne et al. [36], the unacceptable MSI studies yielded T_c_ cooling rates of 0.04–0.06 °C min^−1^. While MSI resulted in a modest T_c_ cooling rate of ~0.01 °C min^−1^, the passive rest condition of Hausswirth et al. [35] resulted in a T_c_ increase of ~0.7 °C, highlighting residual heat production due to rapid transition from exercise to cooling protocol as a possible confounder. The cooling rate reported by Dunne et al. [36] appears to be a conservative estimate as T_c_ decreased a further ~0.8 °C following cessation of cooling. Excluding Peiffer et al. [40], the MSI studies that resulted in unacceptable cooling rates were characterised by minimum immersion duration of 15 min [2, 30, 35–41]. Further, T_c_ of participants prior to cooling was less than 38.8 °C for all treatments excluding [41]. Nine of the unacceptable MSI studies employed 8–15 °C water temperatures, three studies utilised temperate water of 20–26 °C [2, 35, 41] and only one of the 12 studies circulated water [41]. A substantial delay (25 min) between exercise cessation and immersion was reported in four treatments [39, 40].

Ambient temperatures of the MSI studies ranged from 18 to 29.5 °C, whereas six HFI treatments were conducted in 31 °C or greater [4, 15, 20, 21]. In general, the HFI studies (Table 4) were conducted on firefighters who wore t-shirt and open bunker pants while undertaking HFI in reservoirs [4, 15, 56–58] or commercially available chairs or similar [3, 14, 19–21]. Selkirk et al. [4] was the sole HFI investigation to circulate water. With the exception of pre-treatment T_c_ greater than 38.8 °C [4, 58], participants of HFI studies demonstrating unacceptable cooling rates commenced cooling with mean T_c_ of 38.2–38.5 °C. Thus, HFI pre-treatment T_c_ was generally lower than for those studies resulting in acceptable or ideal cooling rates, but similar to the unacceptable MSI treatments. Excluding Khomenok et al. [57], a minimum of 15 min HFI was undertaken. Water temperatures for HFI were broadly classified as 10–15 °C [19, 20, 56–58] or ~17 to 24.9 °C [3, 4, 14, 15, 21, 55].

**Table 4:** Descriptive data for HFI studies meeting the inclusion criteria

Cooling rate	n	Age (years)	T_a_ (°C)	Mins to treatment	T_w_ (°C)	Duration (mins)	BMI (kg m^2^)	BSA (m^−2^)	T_c_ site	Pre T_c_	Δ T_c_ (°C min^−1^)	References
Unacceptable	7NR	40.0	18.0	NR	19.0	15	28.2	2.1	GI	38.2	~0.01	[14]
	10M	22.6	NR	5	17.0	20	27.4	2.1	R	38.5	~0.02	[55]
	15M	40.7	35.0	5	17.4	20	26.5	2.1	R	38.3	~0.02^a^	[4]
	9M	22.0	31.2	3	16.4–17.8	30	21.5	1.9	R	38.5	~0.02	[15]
	24M/1F	36.5	32.5	0	~10.0	15	25.6	2.0	GI	38.2	~0.03^b^	[20]
	6M	NR	35.0	5	17.4	20	NR	NR	R	38.9	~0.03^a^	[4]
	14NR	29.3	15.0	NR	12.5	20	NR	NR	GI	38.5	~0.04	[56]
	17M	24.0	35.0	0	10.0	10	21.7	1.8	R	~38.2	~0.04	[57]
	10M/2F	30.7	33.3–36.9	0	24.9	20	26.4	2.0	GI	38.5	0.05^b^	[21]
	11NR	32.2	22.5	NR	20.9	30	28.2	2.0	GI	38.3	0.05^b^	[3]
	14 M/4F	25.9	24.0	NR	14.3	20	25.5	1.9	GI	~38.3	~0.05	[19]
	6M/1F	25.0	20.7	NR	12.0	15	23.9	1.9	R	39.0	~0.05	[58]

*n* number of participants, *M* male, *F* female, *T*_*a*_ ambient temperature, *T*_*w*_ water temperature, *R* rectum, *GI* gastrointestinal tract, *NR* not reported

^a^circulated bath

^b^field treatment

Whereas four of the respective ideal and acceptable cooling rate treatments tested participants with mean BMI greater than 25.0 kg m^−2^, a total of 10 cohorts from the unacceptable (seven HFI; three MSI) treatments exceeded this benchmark.

In general, MSI of participants with pre-treatment T_c_ of 38.2–38.8 °C in uncirculated 8–26 °C water for a minimum 15 min, and all HFI studies resulted in unacceptable cooling rates, respectively.

## Discussion

Rapidly dissipating body heat during rest periods is essential to maximising the safety and operational performance of firefighters, and to maintain the capability of fire services to sustain emergency responses. Yet the most practically efficient method of lowering firefighters T_c_ in field settings remains unresolved. The NFPA recommendation of HFI as an active cooling modality [22] preceded its implementation by fire services in North America, South East Asia and Australia. The unacceptable T_c_ cooling rates reported in this review prompt re-evaluation of HFI as a cooling method capable of rapidly lowering firefighters T_c_. The cooling protocols deemed acceptable or ideal by this report exclusively utilised immersion of larger body segments, thereby highlighting the potential of MSI to rapidly lower T_c_ of firefighters following work tasks in the heat. The following sections discuss factors contributing to the efficacy of water based cooling, inclusive of cooling thermodynamics, BSA cooled, duration of cooling, pre-treatment T_c_, and the effect of environmental conditions. Practical considerations for the use of water based cooling of firefighters at the fire-scene are also discussed to yield practical evidenced based guidelines.

### Afterdrop

Prior to considering the implications of the review findings for fire scene rehabilitation, the physiology of water immersion must be considered. The critical analysis of the literature is complicated by T_c_ measurement immediately following immersion, where steep thermal gradients may exist within the body. As a consequence, such data may not account for the ‘afterdrop’ phenomenon, where the redistribution of body heat to cooled tissues post-immersion results in a rapid T_c_ decline. The afterdrop occurs due to counter current (blood to blood), convective (blood to tissue) and conductive (tissue to tissue) mechanisms [59], and is therefore proportional to cooling ‘stimulus’ (water temperature, duration and body surface area cooled). Hence, the afterdrop phenomenon has greater relevance for MSI studies, with cooling rates reported immediately post immersion likely representing conservative estimates of T_c_ cooling where the afterdrop was not accounted for. For example, Dunne et al. [36] utilised 15 min of lower body immersion in 15 °C to reduce mean T_c_ by ~0.5 °C during cooling, and reported a further mean T_c_ decrease of ~0.8 °C from cessation of cooling to the 5th min of subsequent exercise bout. Hence, less than half the total T_c_ decrease occurred during immersion, and the total cooling power was therefore not reflected in the reported cooling rate of ~0.03 °C min^−1^.

### Body surface area cooled

It is intuitive to expect a relationship between the proportion of body surface immersed and T_c_ cooling rates. With the exception of one study [30], ‘neck out’ immersion resulted in T_c_ cooling rates (0.08–0.35 °C min^−1^) that were deemed acceptable or ideal [44, 46, 47, 49, 50]. Should these results be replicated at fire scenes, cooling firefighters from T_c_ of 39.0 °C to a desirable fire scene re-entry T_c_ of less than 38.0 °C would require ~3 to ~13 min. Based upon this analysis, the most effective depth of immersion for returning firefighters to safe T_c_ limits [22, 23] is likely to be ‘neck out’ cooling.

While firefighter rehabilitation should aim to rapidly reduce T_c_ prior to redeployment, the use of cold water immersion on large surface areas may not be well tolerated, and little is known of firefighters ability to return to work in these scenarios. In laboratory settings, the impact of MSI on subsequent physical performance is dependent upon the extent of T_c_ and muscular cooling, and work activities undertaken. Endurance performances generally improve due to reduced T_c_ [32, 45], while high velocity movements exhibit the greatest performance susceptibility to lower muscle temperatures [39, 60]. Acknowledging the reduction of muscle temperature with cold water immersion, Versey et al. [61] recommend that athletes allow a minimum of 45 min from cessation of immersion to performance of high intensity, explosive activities unless an adequate warm up is undertaken. Such a delay is not tenable during an emergency response. While firefighters may be required to undertake some high intensity tasks [62], they are not likely to replicate the demands of elite sport, and are anticipated to commence muscular rewarming through metabolic heat production, muscular perfusion and insulation of skin upon re-application of PPC [63]. Post immersion issues were not reported for firefighters returning to work following two 30 min bouts of temperate water immersion to mid sternum despite lowering mean T_c_ below resting values during the subsequent work phase [2]. If low muscle temperature was identified as limiting firefighter physical performance, exclusively cooling the torso (~35 % BSA) by recumbent cool water immersion (10–14 °C) in a small pool or tub is an alternative that has achieved equivocal results [31, 37]. While additional analysis of this technique is required, such an approach has been used in elite sport settings during brief (10 min) scheduled breaks in play to simultaneously remove body heat and minimise muscle temperature decrement of the lower body [24].

A compromise between ‘neck out’ and torso only immersion is the combination of torso and upper leg cooling (54 % BSA). Three treatments utilised this approach in 1–14 °C water to achieve ideal T_c_ cooling rates (0.16–0.20 °C min^−1^) [32, 33]. The efficacy of this method with temperate water is yet to be reported, however it’s not expected to achieve ideal T_c_ cooling rates. The most commonly applied water depth of studies in this review was immersion to the mid sternum, with mixed results (0.01–0.27 °C min^−1^). Ideal and acceptable T_c_ cooling rates for mid sternum depth immersion were achieved across a broad range of water temperatures (2–23.5 °C), and T_c_ cooling rates were 0.10 to ~0.18 °C min^−1^ when water was circulated. An example of the cooling power conferred by water circulation is presented by a comparison of Gagnon et al. [49] and Proulx et al. [50]. Gagnon et al. [49] reported a cooling rate of 0.14 °C min^−1^, which is less than half the rate achieved by Proulx et al. [50] for comparable water temperature, pre-treatment T_c_ and immersion depth. Unlike Gagnon et al. [49], Proulx et al. [50], circulated water, thereby minimising warming of the boundary layer to maintain the thermal gradient between water and the skin. Thus, where feasible, it is recommended to circulate water during immersions to minimise the required immersion duration. Water circulation is likely to be of greater importance during temperate water immersion, where narrower water to skin gradients extend the required cooling times.

In contrast to ‘neck out’, mid sternum, and torso immersion, HFI relies on cooling applied to a combined body surface area of just 19 %. Yet this small biological mass is thought to be preferential for heat exchange with NFPA 1584 stating “the vascularity of blood vessels close to the skin of the arms and hands acts as an excellent means of heat transfer” [22]. The existence of arteriovenous anastomoses (AVA) are cited within the HFI literature [4, 14, 19] as the rationale for preferential heat transfer. Return of blood flow from the extremities through superficial veins of the forearms is thought to permit superior heat exchange during cooling, due to the higher rate of blood flow compared with the capillaries [64]. Additionally, Taylor et al. [65] highlight the superior surface area to mass ratio of the hands as a complimentary factor supporting heat loss. Yet, it’s apparent from studies examined in this review, that regardless of the perceived advantages presented by AVA’s, HFI lacks cooling power given the unacceptable T_c_ cooling rates to date.

Like the hands and forearms, the shallow arteries of the neck, groin and axilla regions ought to provide for preferential heat exchange. Ice pack cooling of these regions is currently recommended by the Australian Resuscitation Council for heat stroke symptomatic patients [66]. However, as for HFI, cooling of these regions by instant cold packs also produce unacceptable T_c_ cooling rates [67], that can be improved to 0.07 °C min^−1^ through extended application (40 min) of larger ice volumes [68]. Collectively, these reports highlight that brief localised superficial cooling is unlikely to achieve acceptable T_c_ cooling rates in firefighting settings. Use of water less than 10 °C may improve HFI cooling rates, but is likely to negatively impact manual dexterity following immersions of just 5 min [69]. Further, the logistical issues surrounding the provision of cold water, particularly in tropical settings, will minimise the likelihood of adoption by fire services.

### Pre-treatment T_c_ and duration of cooling

Cooling at fire scenes must occur rapidly to allow firefighters to safely re-enter fire affected buildings or redeployment to other operational tasks. Thus employing cooling methods that minimise the required exposure time are of interest to incident controllers and fire services generally. Immersion duration should be considered with T_c_ prior to cooling, as a fivefold increase in duration can produce a threefold decrease in cooling rate [48]. Specifically, where cooling commences at a relatively low T_c_ post work, cooling rates are likely to be lower than those where T_c_ approaches 40 °C. With a minimum pre-treatment T_c_ of 39.5 °C, treatments classified as ideal established a larger temperature gradient between deep tissue and water than all but two of the acceptable, and two of the unacceptable MSI studies, respectively. Thus, it appears that MSI is likely to be most effective when T_c_ is high and time critical cooling is necessary. An interesting observation of HFI research is that T_c_ prior to cooling has exceeded 38.5 °C on just two occasions [4, 58]. Low pre-treatment T_c_ (38.2–38.5 °C) also confounded MSI with cooling rates of ~0.03 to  ~0.09 °C min^−1^ [2, 36, 38, 39, 42, 44, 46], that were generally lower compared with treatments where higher T_c_ was prevalent. Thus, the unacceptable T_c_ cooling rates for HFI could be partially attributed to this observation, as higher T_c_ appears to provide a preferential gradient for heat exchange. Examination of HFI treatments applied to cohorts with higher pre-treatment T_c_ is warranted.

The unacceptable cooling treatments were applied to a greater proportion of cohorts with BMI in the overweight or obese categories (>25.0 kg m^−2^), than those studies reporting ideal and acceptable cooling rates. Higher body mass [70] and lean body mass [41] slow T_c_ cooling rate, requiring colder water and/or extended immersion duration to increase heat transfer. This is an important consideration when implementing fireground cooling, as BMI is likely to be above the normal range [71–73] due, in part to increased muscle mass required to safely complete firefighting tasks. The aforementioned circulation of water during immersion may offset some of the additional immersion time required for high BMI cohorts. Firefighter age and gender are additional considerations for determining optimal cooling strategies. Ageing is associated with deteriorating body composition of firefighters [73], and older individuals demonstrate decreased ability to dissipate body heat during and following physical activity [74, 75]. Disparities in body composition also contribute to gender differences for body heat dissipation [76], potentially placing female firefighters at a thermoregulatory disadvantage to male counterparts. The ageing firefighter workforce [73] and the push to increase the proportion of female firefighters working in urban fire services worldwide [77], highlight the need to expand MSI and HFI research beyond the predominantly young male cohorts examined to date.

Irrespective of firefighter characteristics, effective cooling during rest breaks should manifest in lower T_c_ than achieved by resting in the shade. A lower T_c_ upon fire scene re-entry is likely to permit maintenance of cooler T_c_ during a standardised workload, however this has been infrequently assessed. While this review included 43 MSI treatments, the effect of only seven treatments were evaluated during a subsequent work bout. Each MSI treatment resulted in a lower pre and post exercise T_c_ than control trial during fixed and self paced exercise of 6–45 min [2, 32, 35, 36, 42, 45, 46]. In contrast, five HFI treatments examined a subsequent work bout with indifferent results. Where HFI lowered T_c_ to a greater extent than control trial, a lower T_c_ was maintained throughout the exercise period (20–60 min) [4, 14, 57], albeit to a lesser degree than the MSI treatments. Conversely, Hostler et al. [19] and Zhang et al. [58] failed to lower T_c_ with reference to their respective control groups and reported no T_c_ differences during the following work bout. While additional cooling studies mimicking fireground rehabilitation are required, the T_c_ benefits of MSI prior to subsequent exercise appear to be maintained throughout protocols tested to date.

### Environmental conditions

While highlighting the inability of HFI to lower T_c_ to pre-work levels, and that the highest T_c_ cooling rates had been achieved with reservoirs larger than those provided by commercially available chairs, McEntire et al. [78] declared “HFI is likely the best modality for cooling firefighters in hot, humid conditions”. Hot and humid conditions present a unique challenge for firefighter welfare, by combining the greatest need for cooling with challenging conditions to administer it. Yet, when tested in ambient temperatures exceeding 28 °C, HFI in water temperatures of 10–24.9 °C resulted in T_c_ cooling rates of 0.02–0.04 °C min^−1^ [4, 15, 21, 57, 58]. The relatively slow T_c_ cooling rates reported by these studies, ought to prompt investigation of alternate cooling strategies for time sensitive operations in the heat. By excluding research that did not use thermal or chemical protective clothing during exertion from their analysis, McEntire et al. [78] omitted all 43 MSI treatments reported by the current review, despite recognition of MSI as a valid cooling method for use with heat affected individuals [79]. When MSI was conducted in similar ambient temperatures (>28 °C) and utilised comparable water temperatures (~10–25 °C) to the aforementioned HFI studies, superior T_c_ cooling rates (~0.04 to 0.28 °C min^−1^) were reported [2, 31, 32, 46, 54]. Thus, it is likely that immersion of larger proportions of BSA will minimise the influence of adverse environmental conditions on T_c_ cooling rates, as water is a superior conductor of energy than air [80], clamping skin temperature near that of the water.

In hot operating environments, cold water provision may be a logistical issue, prompting the analysis of temperate water immersion as provided by reticulated supplies. In a tropical setting, Brearley et al. [2] reported that mid sternum depth MSI in 25 °C water produced T_c_ cooling rates of ~0.05 °C min^−1^ over 15 min, and ~0.04 °C min^−1^ over 30 min. Despite the relatively slow cooling rates, MSI resulted in an afterdrop during the initial 10 min of a second work bout post-cooling. Modifying the cooling protocol to increase immersion depth and/or circulating water [81] is likely to facilitate more rapid cooling. This was demonstrated by Taylor et al. [47], who achieved a cooling rate of 0.10 °C min^−1^ for ‘neck out’ immersion in circulated 26 °C water. Additional support for temperate MSI is provided by the acceptable [41, 42] and ideal [48] cooling rates. Thus, on available evidence, temperate MSI confers greater cooling than HFI and is therefore better suited for use by firefighters working in hot and humid conditions.

Based on the T_c_ cooling rates reported by this review, protracted rehabilitation times are anticipated when utilising HFI for post incident cooling. Specifically, utilising the highest cooling rate reported for HFI of ~ 0.05 °C min^−1^ [19], achieving T_c_ reduction from 39.0 °C to less than 38.0 °C within the 20 min recovery period is unlikely (~19 min), particularly when allowing for reconditioning of breathing apparatus and PPC prior to fire scene re-entry. It is possible that higher T_c_ prior to HFI would produce more rapid cooling rates than those reported within this review. However, the setting in which HFI yielded a cooling rate of ~0.05 °C min^−1^, found no benefit for HFI compared to passive rest in temperate conditions (24 °C), use of a hand cooling device, infusion of 4 °C intravenous fluids, fan cooling, or ice vests. In that study, only two of 17 participants (including 13 firefighters) rated HFI as the method that ‘reduced their temperature the most’ [19]. Furthermore, HFI was not rated as the cooling method ‘that felt best’ by any participant, and no participant rated HFI as ‘first choice of cooling on the fireground’ [19]. Such perceptions may contribute to the apparent infrequent use of forearm cooling chairs, despite their availability within USA fire services [20].

### Logistical considerations

Cheung et al. [82] acknowledge the cooling benefits of MSI. However, they conclude, “the logistical difficulties involved with erecting multiple baths and supplying cold water in the field, along with the need to remove and don equipment, limit the feasibility of this technique”. A key factor in the comparison of HFI and MSI is that both techniques require the provision of water to establish reservoirs for cooling. As demonstrated by DeGroot et al. [83] in a military setting, establishing group HFI is comparable to preparing small baths, whereas individual HFI requires discrete buckets or chairs with reservoirs for each arm. Thus, given that both methods require a similar ‘footprint’ at the fire scene and also dedicated rehabilitation staff to monitor and maintain cooling apparatus, there are negligible differences in the logistics of water provision for MSI or HFI.

A benefit of firefighter HFI is the time advantage of not removing boots and pants prior to cooling, permitting additional cooling time during rest periods (estimated at 2–3 min). Yet, wearing PPC during rehabilitation likely slows the rate of cooling during rest periods, and this may contribute to the unacceptable HFI cooling rates. Furthermore, the superior T_c_ cooling rates reported for MSI indicate that any time benefits of only partially removing PPC would likely be overcompensated for by the disparity in time to cool the body. While MSI during brief rest periods may be viewed as time inefficient, implementation of MSI during short scheduled breaks of elite individual [84] and team sport competition [24], highlights the utility of this method in time sensitive field settings.

Establishing firefighter cooling would be expedited by deployment of a purpose built rehabilitation facility to fire scenes. Several recommended schematics exist [2, 22] that permit firefighter rehabilitation in a discrete setting, isolated from public view. Public perception is an important consideration when establishing a fireground rehabilitation sector, as firefighters undertaking HFI or MSI in public view while colleagues are responding to an emergency may be misinterpreted as an inadequate response. Screened sides/walls of the rehabilitation sector or a transportable pod [5] would allow for privacy and negate concerns of inferior public image due to firefighters undertaking cooling. Conversely, visibly compromised firefighters re-entering the fire scene could undermine public confidence.

### Limitations of this review

This review applied the criterion of T_c_ cooling rate to evaluate the efficacy of MSI and HFI treatments following physical exertion, excluding additional physiological variables and perceptual ratings from the analysis. The lack of research systematically contrasting MSI and HFI treatments of the same cohort is a limitation of this field of research and therefore this review. Furthermore, the heterogeneous nature of the MSI research compared to HFI in terms of pre-treatment T_c_ and water temperature, provides a more comprehensive overview of the responses to MSI than HFI. Lastly, there is limited data regarding implementation of MSI and HFI in firefighting settings, with particular reference to performing physical activity post cooling.

### Recommendations for future research

Crossover design studies controlling for heat acclimatisation status, fitness and experience are recommended to evaluate the responses of emergency responders to HFI and MSI. Studying the responses to MSI and HFI of cohorts with varied age categories, inclusive of female participants, would more accurately reflect modern firefighting populations. Evaluation criteria should include well-being and productivity measures in addition to T_c_ cooling rate. Pre-treatment T_c_ should represent substantially elevated T_c_ (~39.5 °C) beyond the narrow range currently tested within HFI research, while conforming to ethical guidelines. The T_c_ afterdrop following HFI and MSI ought to be accounted for during such research, and inclusion of functional movement variables would be beneficial.

### Summary

There are examples of HFI indoctrinated within North American fire department policies and recent examples from Australasia. The adoption of HFI for firefighter rehabilitation seems to be underpinned by the NFPA 1584 recommendation of HFI as an active cooling modality [22], referencing an investigation examining just two cooling interventions, HFI in 17.4 °C water, and seated rest in front of a misting fan compared to a passive control [4]. While HFI was more effective than seated rest in lowering T_c_, the cooling rate was modest (0.02 °C min^−1^), and not evaluated against proven field based cooling methods.

Despite the use of MSI in similarly time sensitive and high pressure environments to those faced by firefighters, including elite sport and medical settings, MSI remains largely untested in firefighter rehabilitation settings. This is a surprising outcome given that MSI in cold water has been recognised as the gold standard for treatment of exertional heatstroke and restoration of function for heat affected individuals for the past ~115 years [85]. Of the two known studies to employ water immersion in firefighter rehabilitation settings, both have demonstrated the utility of MSI for lowering T_c_ when compared with passive cooling methods. Cold water, lower body immersion for 15 min in temperate conditions [5] achieved acceptable T_c_ cooling rates, while 30 min of temperate water immersion to a depth of mid sternum in tropical conditions [2] resulted in unacceptable cooling rates by not accounting for an afterdrop. Further, despite the relatively slow cooling rates reported by Brearley et al. [2], MSI achieved twice the T_c_ cooling reported by Selkirk et al. [4] for HFI in harsh environmental conditions. While HFI is yet to be thoroughly evaluated against MSI in hot settings, pilot testing in hot and humid conditions by the authors identified that alternative cooling modalities were more likely to achieve acceptable T_c_ cooling rates (unpublished observations).

## Conclusions

Based upon the extensive field of research supporting immersion of large body surface areas and comparable logistics of establishing HFI or MSI, it is recommended that fire and rescue management reassess their approach to fireground rehabilitation of responders. Specifically, we question the use of HFI to rapidly lower firefighter core body temperature during rest periods. By utilising MSI to restore firefighter T_c_ to safe working levels, fire and rescue services would adopt an evidence-based approach to maintaining operational capability during arduous, sustained responses. While the optimal MSI protocol will be determined by the specifics of a given response, maximising the body surface area immersed in circulated water of up to 26 °C for 15 min is likely to return firefighter T_c_ to safe working levels during rest periods. Cooler water temperatures will augment T_c_ cooling rates and minimise immersion duration.

## Abbreviations

AVA: arteriovenous anastomoses
BMI: body mass index
BSA: body surface area
HFI: hand and forearm immersion
MSI: multi segment immersion
NFPA: National fire and protection authority
PPC: protective clothing
T_c_: core body temperature
